# Predictors of pathological complete response to neoadjuvant chemoradiotherapy for esophageal squamous cell carcinoma

**DOI:** 10.1186/1477-7819-12-170

**Published:** 2014-05-29

**Authors:** Ren-Wen Huang, Yin-Kai Chao, Yu-Wen Wen, Hsien-Kun Chang, Chen-Kan Tseng, Sheng-Chieh Chan, Yun-Hen Liu

**Affiliations:** 1 Department of Plastic and Reconstructive Surgery, Chang Gung Memorial Hospital, Linkou, College of Medicine, Chang Gung University, Taoyuan, Taiwan; 2 Division of Thoracic and Cardiovascular Surgery, Chang Gung Memorial Hospital, Linkou, College of Medicine, Chang Gung University, Taoyuan, Taiwan; 3 Clinical Informatics and Medical Statistics Research Center, Chang Gung University, Taoyuan, Taiwan; 4 Division of Hematology/Oncology, Chang Gung Memorial Hospital, Linkou, College of Medicine, Chang Gung University, Taoyuan, Taiwan; 5 Department of Radiation Oncology, Chang Gung Memorial Hospital, Linkou, College of Medicine, Chang Gung University, Taoyuan, Taiwan; 6 Department of Nuclear Medicine, Chang Gung Memorial Hospital, Keelung, College of Medicine, Chang Gung University, Taoyuan, Taiwan

**Keywords:** Pathological complete response, Esophageal cancer, Predictors, Neoadjuvant chemoradiotherapy

## Abstract

**Backgrounds:**

In this study, we evaluated the factors associated with a pathologic complete response (pCR) after neoadjuvant chemoradiotherapy (nCRT) for esophageal squamous cell carcinoma (ESCC).

**Methods:**

Pre-nCRT parameters in ESCC patients treated between 1999 and 2006 were analyzed to identify predictors of pCR. All patients received 5-fluorouracil/cisplatin-based chemotherapy and external beam radiation followed by scheduled esophagectomy. Variables were analyzed using univariate and multivariate analyses with pCR as the dependent variable. Estimated pCR rate was calculated with a regression model.

**Results:**

Fifty-nine (20.9%) of 282 patients achieved pCR. Univariate analysis identified four patient factors (age, smoking status, drinking history and hypertension), one pre-nCRT parameter (tumor length) as significant predictors of pCR (all *P* <0.05). On multivariate analysis, tumor length ≤3 cm (favorable, odds ratio (OR): 4.85, *P* = 0.001), patient age >55 years (favorable, OR: 1.95, *P* = 0.035), and being a non-smoker (favorable, OR: 3.6, *P* = 0.003) were independent predictors of pCR. The estimated pCR rates based on a logistic regression including those three predictors were 71%, 35 to approximately 58%, 19 to approximately 38%, and 12% for patients with 3, 2, 1 and 0 predictors, respectively.

**Conclusion:**

Age, smoking habit and tumor length were important pCR predictors. These factors may be used to predict outcomes for ESCC patients receiving nCRT, to develop risk-adapted treatment strategies, and to select patients who could participate in trials on new therapies.

## Background

Carcinoma of the esophagus is a highly malignant cancer and is generally associated with a poor prognosis [1]. Despite significant advances in surgical and anesthetic techniques, numerous patients develop recurrence after an apparently curative resection [1]. In order to improve resectability and reduce distant metastases, a treatment regime combining chemotherapy, radiotherapy and surgery was introduced in 1995 [2]. A recent meta-analysis has confirmed the benefit of this strategy [3]. However, there is growing evidence that neoadjuvant chemoradiotherapy (nCRT) significantly prolongs survival only in patients who have a good response, as measured by a pathological complete response (pCR), where no residual tumor is found on the resected specimen [4-6]. A pCR occurs in approximately 15 to 30% of cases, and approximately 60% of patients in whom pCR is achieved survive beyond five years, irrespective of the actual treatment protocol or tumor histology [4-6]. Conversely, for patients who do not respond to nCRT, the prognosis was even poorer than for patients who received primary surgery [7]. In these cases, valuable treatment time was lost, patients unnecessarily experienced severe CRT toxicity, and may even have lost the opportunity to have potentially curative surgery. Thus, identification of accurate predictors of pCR would be of great value in optimizing outcomes and in the design of clinical trials.

Previous studies have evaluated the use of clinical parameters as potential markers of tumor response to nCRT in esophageal cancer, and in these studies, a lower clinical stage was associated with a higher pCR rate in the esophageal adenocarcinoma group [8]. Other studies have explored the use of molecular and genetic predictors of pCR. In the study of the latter, microarray technology has been used to investigate the predictive genetic signatures in esophageal cancer, thereby generating classifiers capable of high predictive accuracy [9-12]. However, different studies have generated different sets of markers which do not overlap, and which have not been validated in large-scale human studies [9-12]. Other studies that have attempted to identify specific molecular markers have generally focused on the squamous subtype [13,14]. The prognostic impact of these markers was determined indirectly through interactions with other unfavorable pathological descriptors, such as advanced T stage or lymph node metastases, resulting in their exclusion from multivariate analysis.

Given the lack of success of previous studies in identifying useable predictive markers for pCR to nCRT treatment in esophageal squamous cell carcinoma (ESCC), we undertook a thorough analysis based on multiple common clinical parameters in a large number of patients.

## Methods

### Patients

The medical records of 282 esophageal cancer patients who underwent esophagectomy after nCRT were obtained from the institutional review board-approved database from the Chang Gung Memorial Hospital (CGMH) between 1995 and 2012.

Pretreatment staging involved computed tomography (CT) of the chest and abdomen, esophagography and endoscopic ultrasonography (EUS). Pretreatment tumor length was defined as the maximum tumor length measured using a barium contrast agent. The date of the last follow-up was 31 August 2013. Staging was performed according to the American Joint Committee on Cancer (AJCC) 7th edition.

The smoking status of each patient was classified into two categories: never-smokers and ever-smokers (former and current). Similarly, drinking status was also classified into two groups: never-drinkers and ever-drinkers (former and current).

### Neoadjuvant CRT and surgery

The nCRT regimens were as follows. 5-Fluorouracil (5-FU) was administered as a continuous infusion over 96 h (1,000 mg/m^2^ per day) from Day 1 to Day 4 and from Day 29 to Day 33. Cisplatin was administered as an intravenous bolus (75 mg/m^2^) on Day 1 and Day 29. Radiation therapy between Day 4 and Day 29 consisted of a total dose of 30 Gy in 200 cGy daily fractions, administered five days a week. Preoperative radiotherapy encompassed the whole esophagus together with regional lymphatic tissue. The supraclavicular fossa, celiac and pericardial lymphatic regions were also irradiated, unless the dose delivered to normal tissues was poorly tolerated. Radiation was delivered through paired anterior and posterior treatment portals, and the dose was prescribed to the field center.

Restaging was performed between four and six weeks after completion of nCRT. Criteria for surgical eligibility included the following: (i) the patient was physiologically fit for surgery, with no liver cirrhosis (>Child B) or heart failure (New York Heart Association class III, IV); (ii) no evidence of a tracheoesophageal fistula; and (iii) no recurrent laryngeal nerve invasion. The standard surgical approach consisted of a limited thoracotomy on the right side and intrathoracic gastric tube reconstruction (the Ivor-Lewis procedure) for lesions of the middle/lower-third of the esophagus. Upper-third/cervical lesions were treated with neck anastomosis (the McKeown procedure). Two-field lymph node dissection was performed in all patients, and cervical lymphadenectomy was performed in selected patients who showed evidence of residual disease in the neck. Pyloroplasty and feeding jejunostomy were not routinely performed. A nasogastric tube was placed in each patient until the anastomotic sites were closed, as assessed by esophagography on postoperative Day 14.

Hospital mortality was defined as any death, regardless of cause, occurring (1) within 30 days after surgery in or out of the hospital, and (2) after 30 days during the same hospitalization subsequent to the operation.

### Post-therapy surveillance

After surgery, no adjuvant therapy was given once pCR had been achieved. All patients underwent chest radiography every three months and CT every six months during the first two years. Panendoscopy was performed if symptoms of recurrent cancer were present. Follow-up data were obtained from medical records and referring physicians, and survival data were updated every six months. The National Cancer Registry Database of Taiwan was used to update missing follow-up information.

### Data analysis

Overall survival (OS) was calculated from the date of surgery to the date of death. Disease-specific survival (DSS) was measured from the date of surgery to the date of known cancer-related death. Pathologic CR was defined as the absence of any tumor cells in the operative pathologic specimen, at the primary site and in lymph node regions. Descriptive statistics were used to describe the baseline characteristics, including demographics and pre-nCRT factors of pCR, non-pCR and overall samples. Chi-square tests were performed to determine significant univariate predictors of pCR. All identified significant continuous predictors in univariate analysis were further categorized as corresponding dichotomous variables and each cutoff value was determined by implementing a corresponding binary recursive partition in a conditional inference framework [15]. The criterion in a conditional inference framework is to maximize the 1 minus *P*-value. These were implemented in a non-commercial statistical software R (http://www.R-project.org) with an optional package AUCRF (Random Forest and the Area Under the Curve) and party.

Next, a multivariate logistic regression analysis was then used to identify the significant multivariate predictors of pCR. In a backward, stepwise fashion, the univariate variable (*P* <0.1) with the least significance was eliminated from the multivariate model. This was continued until only significant variables remained. The final results of a multivariate logistic regression were expressed in odds ratios and confidence intervals. Finally, sstimated pCR rates were obtained by the logistic regression using all possible combinations of significant predictors. Survival curves were plotted using the Kaplan-Meier method. Two-tailed *P*-values <0.05 were regarded as significant. All statistical analyses were performed using SPSS 12.0 software (SPSS Inc., Chicago, IL, USA).

## Results

### General characteristics of the study participants

Over the study period, 313 stage II to IV esophageal cancer patients received nCRT followed by surgery in the CGMH. We excluded histology other than squamous cell subtype (n = 31), and 282 patients were finally included in the study. The general characteristics of the entire cohort were summarized in Table 1. There were 272 males and 10 females with the mean age of 55.2 years (range: 31 to 78 years). Most of the tumors occurred in the middle-third of the esophagus (58.4%, 164/282). The pretreatment mean tumor length was 6.79 cm (range: 1.5 to 16 cm), as assessed by esophagography. Fifty-nine (20.9%) of the 282 patients achieved pCR.

**Table 1:** Demographic and pre-nCRT clinical factors in pCR and non-pCR groups

**Demographic factors**				
**Parameters**	**All (n = 282)**	**pCR (n = 59)**	**Non-pCR (n = 223)**	***P*-value**
Gender				0.94
Male	272	57	215	
Female	10	2	8	
Age	55.2 ± 9.8	58.6 ± 9.5	54.4 ± 9.7	0.003
BMI		22 ± 3.3	21.5 ± 3.5	0.3
HTN				<0.001
Yes	28	13	15	
No	254	46	208	
DM				0.093
Yes	16	6	10	
No	266	53	213	
Smoking				<0.001
Ever	254	46	208	
Never	28	13	15	
Alcohol				0.03
Ever	244	46	198	
Never	38	13	25	
Betal nut chewing				0.39
Ever	119	22	97	
Never	163	37	126	
**Pre-nCRT factors**				
**Parameters**	**All (n = 282)**	**pCR (n = 59)**	**Non-pCR (n = 223)**	***P*-value**
Pre-nCRT Hb (g/dl)	13.2 ± 1.66	13.1 ± 1.67	13.2 ± 1.65	0.55
Pre-nCRT WBC (/ul)	8,046 ± 2,967	8,286 ± 3,698	7,982 ± 2,748	0.486
Pre-nCRT Albumin (g/dl)	3.99 ± 0.48	4 ± 0.5	3.98 ± 0.48	0.6
Pre-nCRT Platelet (1,000/ul)	278 ± 98	261 ± 77	283 ± 103	0.15
Tumor length (cm)	6.8 ± 2.6	5.9 ± 2.6	7 ± 2.5	0.003
Tumor grade				0.08
WD	14	3	11	
MD	199	34	165	
PD	69	22	47	
Clinical stage				0.29
II	71	15	56	
III	202	44	158	
IV	9	0	9	
Clinical N status				0.8
No	40	9	31	
Non-No	242	50	192	
LN metastases site				0.15
Non or Mediastinal	236	53	183	
Outside mediastinal	46	6	40	

BMI, Body mass index; Hb, Hemogobin; HTN, Hypertension; nCRT, Neoadjuvant chemoradiotherapy; pCR, Pathological complete response; WBC, White blood cells.

LN,Lymph node; DM, Diabetes mellitus.

The Ivor-Lewis procedure was used in 231 individuals and 48 patients were treated with the Mackeown procedure. Three patients received exploratory surgery without resection due to intraoperatively identified T4 or M1 disease. For reconstruction, the stomach was used in 272 cases and colon interposition in 7 cases. In-hospital mortality occurred in 16 cases.

### Survival outcome: pCR versus non-pCR

**Figure 1:**
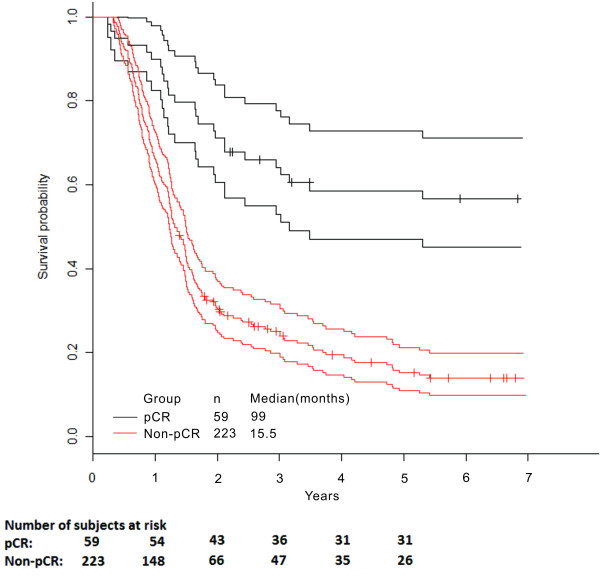
**Overall survival and 95% confidence interval in patients with and without pathological complete response.**

The median OS of the entire population was 18 months (95% confidence interval (CI): 16.5 to 20 months). At the end of the study period, 50.3% of pCR patients and 83% of non-pCR patients had died. The median OS was 98.8 months (95% CI: 43.7 to 153 months) in pCR patients and 15.5 months (95% CI: 13.8 to 17.2 months) in non-pCR patients. This difference was statistically significant (Figure 1, *P* <0.001).

### Univariate analysis of predictive parameters for pCR

The baseline demographic features differed significantly between groups. As shown in Table 1, the pCR group of patients were on average older, fewer had a history of smoking or alcohol use and more had a history of hypertension (HTN). With respect to the pre-nCRT parameters, patients in the pCR group had a significantly shorter tumor length on average. There were no differences in hemoglobin (Hb) levels, white blood cell (WBC) counts, platelet counts or albumin levels before nCRT. Celiac or lower neck lymph node metastases were more common in the non-pCR group with borderline significance (*P* = 0.15).

### Multivariate analysis of predictive parameters for pCR

**Figure 2:**
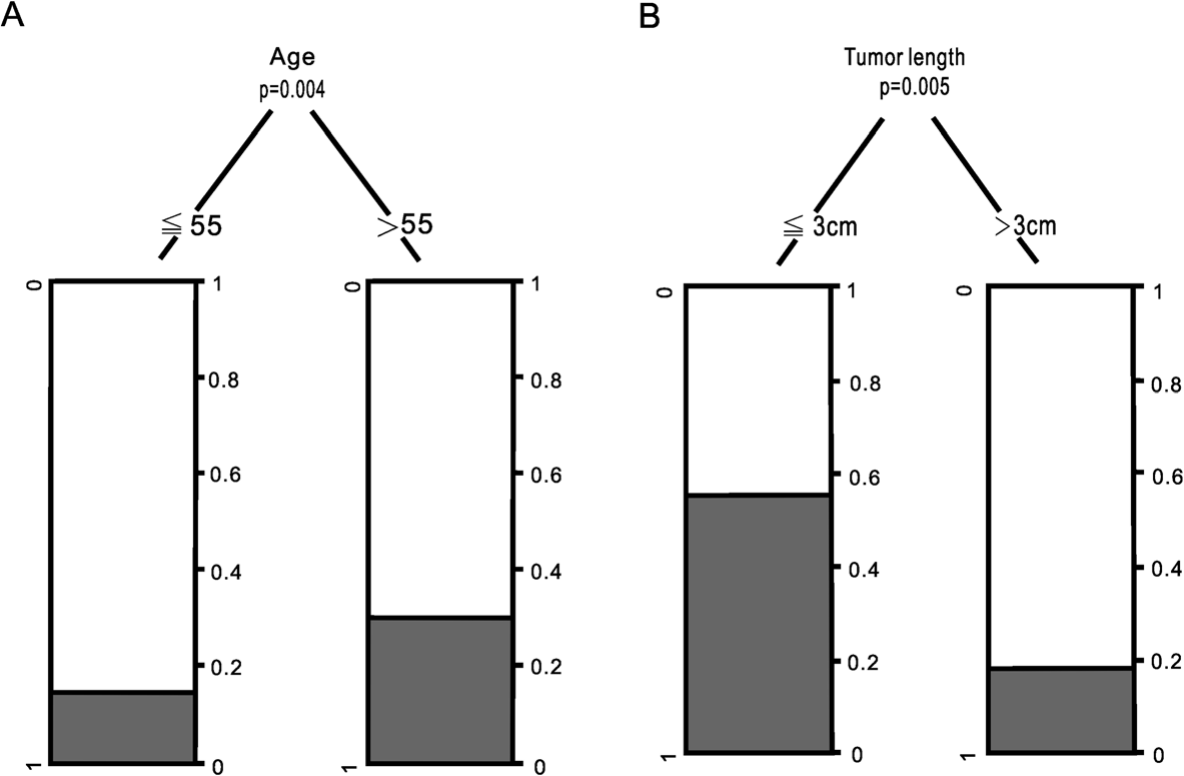
Relative frequencies of pCR and non-pCR groups of the binary classification tree for age (A) and tumor length (B).

In order to improve clinical utility, significant continuous variables selected from univariate analysis (age and tumor length) were further transformed onto an ordinal scale before entering them into a multivariate analysis based on a recursive partition.The optimal cut-points were 55 for age and 3 for tumor length, respectively (Figure 2A, B).

As shown in Table 2, multivariate analysis identified a tumor length of ≤3 cm (favorable, odds ratio (OR): 2.1, *P* = 0.02), patient age of >55 years (favorable, OR: 2.3, *P* = 0.008) and a non-smoking status (favorable, OR: 3.36, *P* = 0.003) as independent predictors of pCR while HTN and alcohol use were eliminated. Finally, the estimated pCR rates based on a logistic regression including those three dichotomous predictors were 71%, 35 to approximately 58%, 19 to approximately 38%, and 12% for patients with 3, 2, 1 and 0 predictors, respectively.

**Table 2:** Multivariate analysis of significant predictors for pathological complete response

**Predictors**	**OR (95% CI)**	***P*-value**
Ever-smoker	Reference	0.003
Never-smoker	3.6 (1.55 to approximately 8.34)	
Tumor length >3 cm	Reference	0.001
Tumor length ≦3 cm	4.85 (1.84 to approximately 12.76)	
Age ≦55 y/o	Reference	0.035
Age >55 y/o	1.95 (1.05 to approximately 3.64)	

## Discussion

Neoadjuvant CRT is currently in widespread use as the first-line treatment for locally advanced esophageal cancer [3]. It has become increasingly evident that only patients with a good response (pCR or near pCR) after nCRT have a survival benefit, whereas for those with little response after nCRT, survival is invariably poor [4-7]. In non-responders, a valuable therapeutic window may have been lost, the patient may have unnecessarily experienced severe CRT toxicity, and may even have lost the opportunity to have potentially curative surgery [7]. Thus, reliable predictive factors for pCR are of great clinical importance. In the current study, we have analyzed a number of potential predictive factors for pCR in esophageal cancer of the squamous cell carcinoma subtype, treated using a uniform CRT protocol. Although we did not evaluate molecular markers, our study is distinguished by its large size and the incorporation of multiple common clinical parameters. We identified three predictive factors for pCR - age, smoking status and tumor length - all of which are easily measureable and, hence, easily verifiable in large-scale trials. These factors may ultimately form the basis for a practical predictive marker for routine clinical use.

Smoking is a well-known risk factor for esophageal cancer [16]. However, few reports directly evaluate smoking as a prognostic factor for esophageal cancer, or determine whether it can influence the response to CRT [17,18]. In our study, we found that smoking was a statistically significant, negative predictor for pCR. Although the mechanism by which smoking reduces the efficacy of CRT is still unknown, overexpression of DNA repair enzymes because of smoking is one possibility. Increased expression of a DNA repair enzyme was reported in heavy smokers with esophageal cancer or non-small cell lung cancers and was also associated with a poor response to CRT [19,20]. Taken together, these reports might explain the poor response to CRT in patients with a heavy smoking habit in our study.

The tumor length in esophageal cancer has long been regarded as an important prognostic factor and is consequently one of the T stage descriptors (<5 cm: T1; >5 cm: T2) in the fifth AJCC edition. However, the importance of tumor length was outweighed by the depth of invasion from 1987 onwards because of their strong inter-correlation [21]. A growing number of studies since then have re-confirmed that tumor length is an indicator of more aggressive behavior independent of T stage [22,23]. Our findings suggest that tumor length might also predict the response to CRT, an observation that could potentially be explained by current radiobiological models, the number of cells killed, as described previously in a study on rectal cancer [24].

Old age was not a significant prognostic factor for esophageal carcinoma in most studies but was identified as a predictor for pCR in the current study [25]. Older patients (>55 years) were 1.95 times more likely to achieve pCR than younger patients (≤55 years) after nCRT. The reasons for this are unclear and may relate to a complex set of interactions. In Taiwan, earlier age-onset esophageal cancer represents a unique cohort; these patients are more frequently associated with habitual use of substances (tobacco, alcohol or areca nut (seed of the Areca palm)) that are known to reduce the response to radiotherapy [26]. In the current study, we also observed a similar trend of habitual use of substances at a younger age (Additional file 1: Table S1).

Predictive factors can be useful in the design of clinical trials for new therapies and, to a lesser extent, to avoid ineffective therapy. For example, in a cohort where no positive predictor was available, only 12% could achieve pCR following standard 5FU/cisplatin-based CRT. These patients might be offered a chemotherapy regimen other than 5FU/cisplatin or received direct surgery whenever curative resection was possible. On the other hand, in patients with multiple predictors, in addition to the routine use of CRT as the first-line treatment, the anticipated high pCR rate also can lead to the implementation of a trial with organ preservation strategy after nCRT.

We are aware that some weaknesses are inherent in this study. These include the fact that this study was retrospective with a long study period and that it did not incorporate any molecular markers or gene expression profiles. In addition, the radiation dose used in nCRT was lower than that currently used in routine practice (30 Gy vs. 45 to 50.4 Gy), which may have contributed to a lower pCR rate. Future validation of our findings with larger sample sizes and different CRT protocols is therefore required.

## Conclusion

In this study, we showed that older patients (>55 years) who have never smoked and with an initial tumor length of ≤3 cm were more likely to achieve pCR. These factors may be used to predict outcomes for ESCC patients receiving nCRT, to develop risk-adapted treatment strategies, and to select patients who could participate in trials on new therapies.

## Abbreviations

AJCC: American Joint Committee on Cancer; CGMH: Chang Gung Memorial Hospital; CT: Computed tomography; ESCC: Esophageal squamous cell carcinoma; Hb: Hemoglobin; HTN: Hypertension; nCRT: Neoadjuvant chemoradiotherapy; OS: Overall survival; pCR: Pathologic complete response; WBC: White blood cells; DM: Diabetes mellitus.

## Competing interests

All authors declare no conflicts of interest.

## Authors' contributions

YKC had full access to all of the data in the study and takes responsibility for the integrity of the data and the accuracy of the data analysis. RWH and YKC contributed to the conception and design of the study, data acquisition, analysis and interpretation of the data, and the writing and revision of the manuscript. YWW contributed to the data analysis and statistical analysis. HKC, CKT, SCC and YHL contributed to quality control of the data and the manuscript review. All authors read and approved the final manuscript.

## Supplementary Material

Additional file 1: Table S1 Demographic and clinical characteristics of patients with different age (≤55 or > 55 years). Click here for file
