# Supplementary material for: Predictors of pathological complete response to neoadjuvant chemoradiotherapy for esophageal squamous cell carcinoma
Source: World J Surg Oncol. 2014 May 29;12:170. doi: 10.1186/1477-7819-12-170 (PMC4050419; doi:10.1186/1477-7819-12-170)
Supplement: Additional file 1: Table S1 — Demographic and clinical characteristics of patients with different age (≤55 or > 55 years). [file 1477-7819-12-170-S1.doc]

Supplemental table 1

Demographic and clinical characteristics of patients with different age (≦55 or >55)

|  | Age ≦55  (n=146) | **Age>55**  **(n=136)** | *P* value |
| --- | --- | --- | --- |
| Gender  Male  Female | 142  4 | 130  6 | 0.45 |
| Smoking  Ever  Never  Drinking  Ever  Never  Betal nuts chewing  Ever  Never  Tumor length (cm) | 136  10  132  14  77  69  6.5±2.9 | 118  18  112  24  42  94  6±1.9 | 0.07  0.048  <0.001  0.1 |
